# The Role of Human Adult Peripheral and Umbilical Cord Blood Platelet-Rich Plasma on Proliferation and Migration of Human Skin Fibroblasts

**Published:** 2017-05

**Authors:** Seyedeh-Sara Hashemi, Mahdokht Mahmoodi, Ali Reza Rafati, Farzad Manafi, Davood Mehrabani

**Affiliations:** 1 Burn and Wound Healing Research Center, Shiraz University of Medical Sciences, Shiraz, Iran;; 2 Division of Pharmacology and Pharmaceutical Chemistry, Sarvestan Branch, Islamic Azad University, Sarvestan, Iran;; 3 Stem Cell Technology Research Center, Shiraz University of Medical Sciences, Shiraz, Iran

**Keywords:** Umbilical cord blood, Platelet rich plasma, Proliferation, Migration, Skin fibroblast

## Abstract

**BACKGROUND:**

Wound healing is a complex and dynamic process following damage in tissue structures. Due to extensive skin damage caused by burn injuries, this study determined the role of human adult peripheral and umbilical cord blood platelet-rich plasma on proliferation and migration in human skin fibroblasts.

**METHODS:**

Platelet-rich plasma (5, 10, 15, 20 and 50% PRP) from human umbilical cord blood and adult peripheral blood were provided and added to fibroblasts cultured from a human skin sample. Migration and proliferation of fibroblasts were assessed in comparison to 10% FBS and by the fibroblast responses to a concentration gradient.

**RESULTS:**

All components of the umbilical cord blood PRP significantly stimulated the growth of fibroblasts when compared to the negative control. Fibroblast growth was enhanced in a dose dependent manner. All fibroblast cultures retained normal morphology. No significant difference was noted between umbilical cord blood and adult peripheral blood PRP preparations regarding cell proliferation and migration, but the difference to 10% FBS was significant. 1% and 50% PRP reduced cellular proliferation. The 20% umbilical cord blood PRP and 10% adult peripheral blood PRP had a significant stimulatory effect on the migration of the skin fibroblast cells in comparison with 10% FBS.

**CONCLUSION:**

As PRP could promote the migration and proliferation of dermal fibroblasts, it can be safely added in cultures when treatment of chronic wounds without triggering the immune response is needed.

## INTRODUCTION

Tissue regeneration is beginning with degranulation of platelets and clotting leading to the release of various cytokines, clotting factors and inflammatory response.^1^ Since growth factors play an important role in the repair process, the use of platelet-rich plasma (PRP) is of great importance.^2^ Platelets naturally stimulate the secretion of growth factors that can initiate the physiological healing process in acute injuries. PRP is widely used as a source of growth factors not only for hard and soft tissue regeneration, but also in plastic surgery.^3^

Human umbilical cord blood (HCB) has great potential to be adopted for research and therapeutic purposes.^4^ Human umbilical cord blood Platelet-rich plasma (PRP) contains increased levels of growth factors (GF), including epidermal growth factor (EGF), vascular endothelial growth factor (VEGF), fibroblast growth factor (FGF), insulin-like growth factor-1 (IGF-1), interlukins and interferons, which are used for growth, proliferation and differentiation of cells in fetal blood.^5^ Other constituents of HCB include serum albumin, tansferrin and fibronectin, mineral , ghrelin, adiponectin, vitamin A and E and several essential fatty acids.^6^^,^^7^

PRP plays an important role in the repair process for many types of cells, such as osteoblasts, fibroblasts, epithelial cells, endothelial cells and adult mesenchymal stem cells.^2^^,^^8^^,^^9^ Fibroblasts are critical in supporting normal wound healing, involved in key processes such as breaking down the fibrin clot, creating new extra cellular matrix (ECM) and collagen structures to support cells associated with wound healing, as well as contracting the wound.^10^ Lam and co-workers have used cord blood plasma for the expansion of cord blood cells and showed that expansion of the megakaryocytic lineage was consistently higher, but reduced CD34+ cells.^11^ Cord blood plasma has also been used to culture T cells for adoptive immunotherapy.^12^

However, to the best of our knowledge, a direct comparison of human umbilical cord blood and adult peripheral blood PRP on cellular proliferation and migration has not been reported in the literature. The aim of this study was to evaluate the effect of PRP on the important wound healing–related cell functions of proliferation and migration and to determine whether there is any difference in the effect elicited by human umbilical cord blood and adult peripheral blood PRP.

## MATERIALS AND METHODS

Primary cultures of fibroblasts were initiated from the full-thickness human skin sample (n=3) derived from cosmetic surgery that were trransferred to the laboratory. The samples were washed three times to remove any probable infection. The skin sample was then cut into 2-3 mm pieces and put in 0.25% trypsin (Gibco Life Technologies) at 4˚C overnight to separate the epidermal layer from the dermis. The dermis was cut into very small pieces (5 mm^2^) using curved scissors and the pieces were transferred into a collagenase Type II (200 IU/ml) solution in an incubator at 37°C for 2 h. 

The suspension was centrifuged at 500 ×g for 5 min. The pellet was resuspended in culture medium. The isolated cells were counted using hemocytometer and seeded into culture dishes at cell density of 2×10^5^ cells/cm^2^. The cells were cultured in a CO2 incubator at 37°C and the culture medium was changed twice a week. When the cells’ density adhered to the bottom of the flasks and reached to 70-80 confluence, cell passaging was done using 0.25% Trypsin-EDTA solution. Cells from passages 3 and 4 were used in the study. 

Platelet-rich plasma was provided from whole adult peripheral or umbilical cord blood collected in lithium heparin–coated tubes and initially centrifuged at 350 g for 10 min to separate the red blood cells (RBC) from the platelet-rich plasma. The upper layer of the RBC portion was the platelets containing the largest amount of growth factors, and hence having the greatest potential of biological activity, that was mixed with the upper 1 mm of RBCs. The inclusion of this small RBC layer imparted a red tinge to the PRP. The PRP portion was then extracted and centrifuged again at 1000 g for 10 min. Dilutions of the PRP (5, 10, 15 and 20%) for the various experiments were produced by diluting with standard serum-free media. 

Proliferation of the skin fibroblasts was assessed using the total cell number by Colorimetric (crystal violet) proliferation assay. The treatment media used in these studies were serum-free media (negative control), 10% FBS (positive control), 5, 10, 15, 20 and 50% PRP. As described earlier, all dilutions were prepared with serum-free media. The viability of cells was determined by MTT assay. Approximately 1×10^5^ cells were transferred to 96-well plate and incubated for 24 h at 37°C, and treated with different concentration of PRP. The volume of the well was 100 micro liters. 

Micro plates containing cell extract for 24h, were incubated in the same conditions. A total of 10 ml solution of MTT (5 mg/ml) was added to each well and was incubated for 3 hours. One-hundred milliliter of DMSO was replaced with incubated MTT medium. Then the optical absorbance was measured at a wavelength of 570 nm with ELISA reader. Viability percentage of cells that were affected by different concentration of the PRP was calculated by dividing the absorbance of treated wells to the absorbance of control well and then multiplied by 100. The results (mean±SEM) were expressed using SPSS software (version 12, Chicago, IL, USA). 

The migration assay was carried out using Trans well Permeable with a 6.5-mm diameter, 8.l m pore size, as previously described.^13^ Briefly, migration from one side of the membrane to the other was examined after 6 h in the presence of six formulations of treatment media– serum-free media (negative control), 5, 10, 15, 20 and 50% PRP. Three hundred micro-litters of treatment media was placed into the lower compartment of a 24 well plate and allowed to incubate for 30 min. Cells were seeded at a concentration of 2×10^3^ cells diluted in 100 ml of media and delivered onto the upper surface of the permeable insert. 

As the PRP preparations had a viscous consistency and settled at the lower part of the well, a concentration gradient was achieved. After incubation for 6 h, the permeable insert was removed, and the outer membrane was carefully wiped dry to remove non-migrated cells. Thereafter, the permeable inserts, containing only the cells that had migrated through the membrane to the inner surface, were placed into fresh well plates containing 300 µl crystal violet for 15 min. The stained cells on the inner surface of the permeable insert were then solubilized with 33% glacial acetic acid, and the absorbance was read in a spectrophotometer at 570 nm.

One-way ANOVA test was used to assess the effect of the various PRP preparations on cell function. To identify the statistically significant differences between the various treatments, post hoc analysis was carried out using SPSS software and the Bonferroni test. Statistical differences between groups were accepted for P-values less than 0.05. 

## RESULTS

The results of the MTT assay of umbilical cord blood PRP were shown in Table 1, regarding various treatment media readings presented as ratios compared to the negative control. All components of the umbilical cord blood PRP significantly stimulated the growth of fibroblasts when compared to the negative control. Fibroblast growth was enhanced in a dose dependent manner. All fibroblast cultures retained normal morphology. The proliferation of human dermal fibroblasts peaked on 24 hour of culture in the presence of 10% or 20% Umbilical cord platelet-rich plasma and decreased in a dose-dependent manner in the presence of 5% or 50% umbilical cord blood PRP. Cell proliferation was few in the serum-free controls, but it markedly increased in the presence of umbilical cord blood PRP. 

**Table 1:** Proliferation of fibroblasts in the presence of the human adult peripheral blood PRP (MTT assay). Concentration of PRP; 10% FBS in the medium as a positive control; medium without any additives as a negative control (C). Results shown are the means of four parallel cultures±SD.

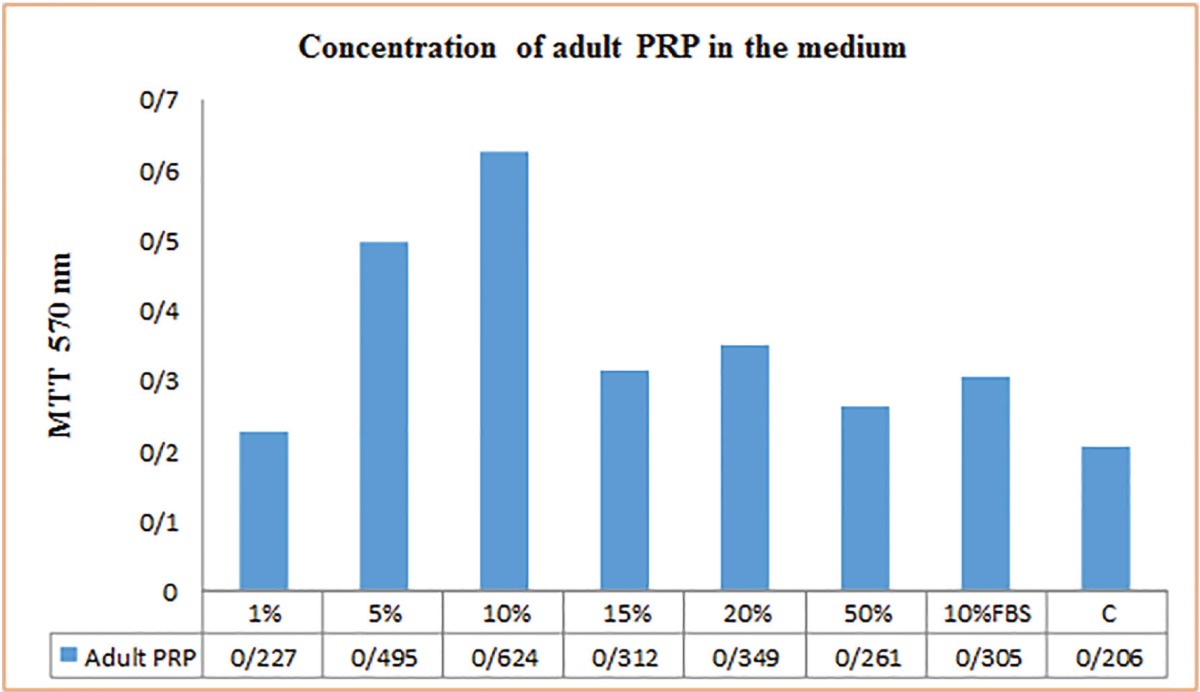

The results of the adult peripheral blood PRP MTT assay were shown in Table 2, demonstrated that during 24 h, adult peripheral blood PRP stimulated cell proliferation when compared to 10% FBS (*p*≤0.05), and all other PRP concentrations (*p*<0.05 for all comparisons). Furthermore, the use of 10% adult peripheral blood PRP resulted in a statistically significant increase in proliferation when compared to10% FBS (*p*≤0.05). When PRP was compared to the positive control of 10% FBS, 1% adult peripheral blood PRP (*p*<0.05) and 50% PRP (*p*≤0.05) exhibited a significant reduction in cellular proliferation, while there was no difference between 10% FBS and PRP (1% and 50%).

**Table 2:** Proliferation of fibroblasts in the presence of the human umbilical cord blood PRP (MTT assay). Concentration of PRP; 10% FBS in the medium as a positive control; medium without any additives as a negative control (C). Results shown are the means of four parallel cultures±SD.

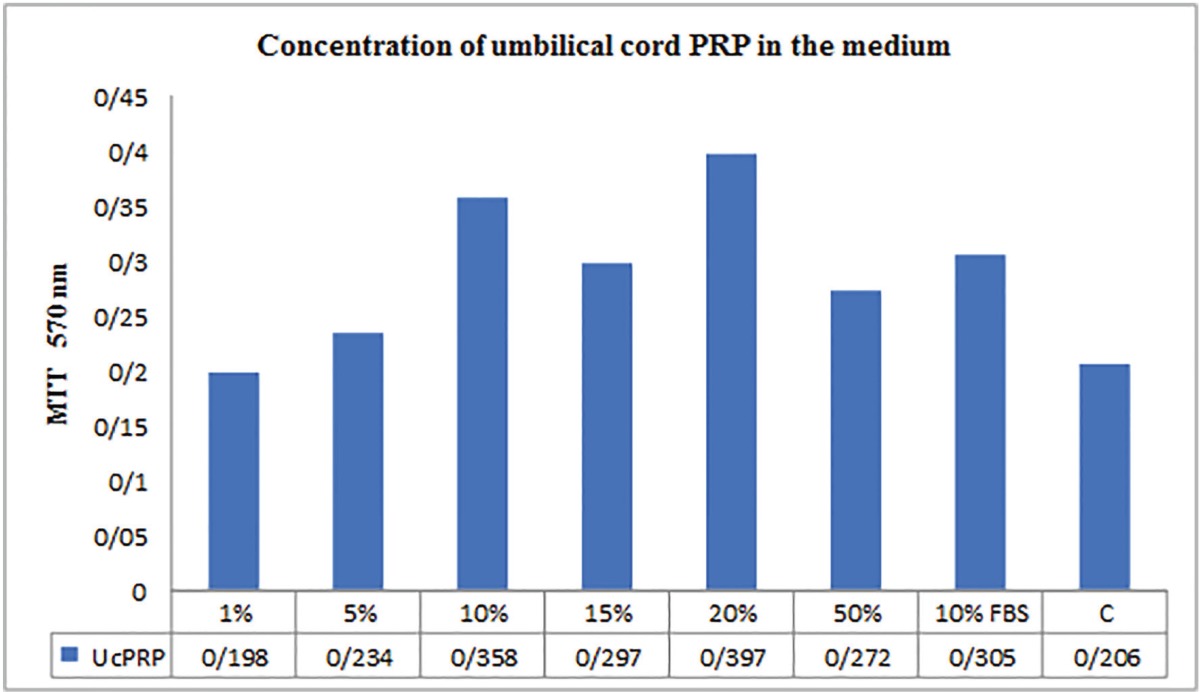

**Fig. 1:**
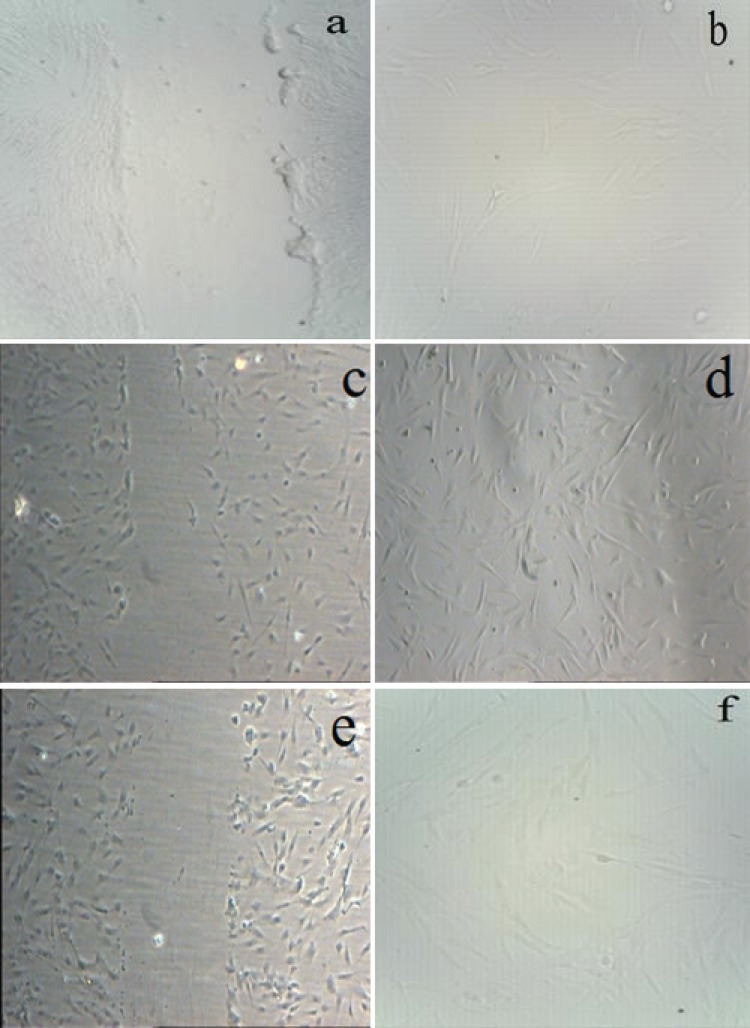
Cell Migration Assay **a.** Control-12 hours, **b.** Control-24 hours, **c.** UC PRP -12 hours, **d.** UC. PRP -24 hours, **e.** A. PRP-12 hours, **f.** A. PRP -24 hours). Cell migration assay shows the movement of fibroblasts (indicated by arrow lines towards the imaginary line drawn in the center of the image) captured at 12 and 24 h after incubation using phase-contrast microscope

The 20% umbilical cord blood PRP and 10% adult peripheral blood PRP had a significant stimulatory effect on the migration of the skin fibroblast cells in comparison with the serum-free media (*p*<0.05). The 5, 15%, 20 and 50% PRP showed a trend toward having a positive effect on migration compared to the negative control, although statistical significance was not reached a significant difference (Figure 1). 

## DISCUSSION

In vitro cell, fibroblast culture play an important role in science, medicine and industry.^14^^-^^17^ We evaluated the PRP potential in proliferation and migration of dermal fibroblasts as a dose-dependent effect of PRP. We showed that PRP had substantial effects on the proliferation and migration of human skin fibroblast *in vitro*. PRP was derived from both adult peripheral blood as the source most often used for its generation, and also from human umbilical cord blood. PRP, when applied directly to human skin fibroblasts, significantly increased fibroblast proliferation and migration which was more for umbilical cord blood PRP as compared to adult peripheral blood PRP, likely due to greater amounts of PDGF and FGF umbilical cord blood PRP. 

PRP is known to contain various growth factors, including PDGF and TGF-β, which play important roles in hard and soft tissue regeneration,^18^^,^^19^ plastic surgery, and sports injuries to regenerate vascular tissue and to serve as a scaffold during post-operative healing.^20^^,^^21^ Beneficial roles of PRP in tissue regeneration have been proposed. In the dental research field, PRP has been studied as a potential means by which to deliver growth factors to a wound healing site to promote tissue regeneration. Moreover, use of PRP prepared from a patient’s own blood will likely reduce harmful immune responses.^22^^,^^23^ It was shown that PRP are used as a source of growth factors that control healing, angiogenesis and promotion of proliferation for fibroblasts, causing differentiation of human dermal fibroblasts into myofibroblasts and promoting wound contraction, and as a potential therapeutic agent for skin wound healing.^24^^,^^25^ Recent evidence suggests that human umbilical cord blood platelet-rich plasma stimulate the proliferation and migration of dermal fibroblasts.^26^

Regarding migration, the results of this study indicated that two concentrations of umbilical cord blood PRP (15 and 20%) significantly stimulated migration of skin fibroblasts. The effect of umbilical cord blood PRP on cell migration has not been widely studied but our findings are consistent with reports that PRP promoted migration of osteoblasts,^13^^,^^27^ periodontal ligament cells,^13^ and skin fibroblasts.^28^ The overall stimulatory effect of PRP on cell proliferation is well established in the literature especially when platelet lysate supernatant is used.^27^^,^^29^ Indeed, these authors showed that PRP-enhanced collagen I synthesis and postulated that the viscous fibrin-rich PRP preparation promoted a relatively complex cell response involving both cell proliferation and differentiation, rather than simply inducing proliferation as seen when only the concentrated platelet lysate supernatant is used. 

It has been shown that different concentration of PRP samples generated from umbilical cord blood PRP contained significantly higher levels of PDGF-AB/BB and FGF-2 with well documented effects on the proliferation of mesenchmal stem cells.^30^^-^^32^ The finding that adult peripheral blood PRP had similar effectiveness to umbilical cord blood PRP that may be important in relation to tissue engineering applications, where PRP has been used to promote cell growth and differentiation.^32^^,^^33^ PRP generated from umbilical cord blood also contained greater amounts of VEGF, a key molecule in the promotion of angiogenesis and neovascularization. In contrast, PRP derived from adult peripheral blood contained more SDF-1, a chemokine demonstrated in multiple previous reports to stimulate chemotaxis of mesenchmal stem cells.^33^

This study compared the effect of umbilical cord blood PRP and adult peripheral blood PRP and showed that there was no difference between these two potential sources. It will be of great interest to determine whether the growth factors and chemokines measured in the current study will account for all of the activities of umbilical cord blood PRP or whether novel biomolecules or factors whose presence has not been reported in cord blood that might also contribute to the greater potency of umbilical cord blood PRP reported herein. Platelets are well documented to contain mitogenic growth factors and molecules that promote tissue repair and angiogenesis. Therefore, in the situation where adult PRP may not be available, umbilical cord blood PRP could be used with the expectation of producing similar effects on cell proliferation and migration needed in wound healing.
